# Unfolding of core nucleosomes by PARP-1 revealed by spFRET microscopy

**DOI:** 10.3934/genet.2017.1.21

**Published:** 2017-01-05

**Authors:** Daniel Sultanov, Nadezhda Gerasimova, Kseniya Kudryashova, Natalya Maluchenko, Elena Kotova, Marie-France Langelier, John Pascal, Mikhail Kirpichnikov, Alexey Feofanov, Vasily Studitsky

**Affiliations:** 1 Biology Faculty, Lomonosov Moscow State University, Moscow, 119992, Russia; 2 Fox Chase Cancer Center, Philadelphia, PA, 19111-2497, USA; 3 Shemyakin-Ovchinnikov Institute of Bioorganic Chemistry of Russian Academy of Sciences, 117997 Moscow, Russia; 4 Department of Biochemistry and Molecular Medicine, Université de Montréal, 2900 Boulevard, Edouard-Montpetit, Montréal, QC H3T 1J4, Canada

**Keywords:** PARP-1 protein, DNA repair, chromatin structure, nucleosome, DNA-histone interactions

## Abstract

DNA accessibility to various protein complexes is essential for various processes in the cell and is affected by nucleosome structure and dynamics. Protein factor PARP-1 (poly(ADP-ribose) polymerase 1) increases the accessibility of DNA in chromatin to repair proteins and transcriptional machinery, but the mechanism and extent of this chromatin reorganization are unknown. Here we report on the effects of PARP-1 on single nucleosomes revealed by spFRET (single-particle Förster Resonance Energy Transfer) microscopy. PARP-1 binding to a double-strand break in the vicinity of a nucleosome results in a significant increase of the distance between the adjacent gyres of nucleosomal DNA. This partial uncoiling of the entire nucleosomal DNA occurs without apparent loss of histones and is reversed after poly(ADP)-ribosylation of PARP-1. Thus PARP-1-nucleosome interactions result in reversible, partial uncoiling of the entire nucleosomal DNA.

## 1. Introduction

Eukaryotic genome is composed of nucleosomes that consist of 145–148 bp DNA segments wrapped around the histone octamer in 1.65–1.7 superhelical coils. Nucleosomal organization limits DNA accessibility to various proteins, including protein complexes involved in DNA repair [1]. Various protein complexes, including ATP-dependent chromatin remodelers and PARP-1 protein reorganize chromatin, making it more accessible to other DNA-interacting proteins.

PARP-1 is an abundant multi-domain protein, localized in cell nuclei of higher eukaryotes, with a range of diversity functions, playing role in DNA repair [2],[3], chromatin organization and transcription [4]. One of the crucial roles of the protein in a cell is detection of DNA damages through its DNA-binding zinc-finger domains that recognize single- and double-strand DNA breaks [5]. A variety of factors (e.g., ionizing radiation) cause genome damage making double-strand breaks in DNA, which can lead to mutations. PARP-1 binding to a DNA strand break induces a conformational change in the protein [6],[7], which results in its DNA-dependent activation and poly(ADP)-ribosylation (pADP-r) of the target proteins (including automodification of PARP-1) using NAD^+^ as a substrate. Core histones [8] and linker histone H1 [9] are among the targets for pADP-r. Some direct inhibitors of PARP-1 enzymatic activity are important anticancer compounds. Thus anticancer compound olaparib interferes with essential nuclear processes in various tumors and causes cell death due to synthetic lethality [10],[11].

Although PARP-1 can bind to intact nucleosomes and to a variety of nucleosome substrates through double-strand break in nucleosomal DNA with different affinities [12], it is unknown whether it affects the structure of the nucleosome core. Using a single-particle Forster resonance energy transfer (spFRET) approach [13]–[16], we report that PARP-1 causes a considerable nucleosome unfolding *in vitro* that can be almost completely reversed by its automodification.

## 2. Materials and Methods

### 2.1. Protein purification and DNA templates

Human recombinant PARP-1 was expressed in *E.coli* and purified as described [17].

Fluorescently labeled DNA templates used for nucleosome assembly were synthesized by PCR using modified nucleosome-positioning sequence s603-42 [18] as a template. The following oligonucleotides were used to introduce fluorescent labels in nucleosomal DNA: for nucleosomes N13/91: forward – 5′-ACCCCAGGGACTTGAAGTAATAAGGACGGAGGG CC**T#**CTTTCAACATCGAT-3′ (Т# refers to a nucleotide with a Cy3 label), reverse – 5′-CAAGCG ACACCGGCACTGGGCCCGGTTCGCGCTCCCTCCTTCCGTGTGTTGTCGT*CTCT-3′ (T* refers to a nucleotide with a Cy5 label). For nucleosomes N35/112: forward – 5′-AAGCGACACCG GCACTGGGCCCGGTTCGCGC**T#**CCCGCCTTCCGTGTGTTGTCGTCTCTCGGGCGT-3′, reverse – 5′-ACCCCAGGGACTTGAAGTAATAAGGACGGAGGGCCTCTTTCAACATCGATGC ACGGT*GGTTAG; for N57/135: forward – 5′- ACACCGGCACTGGGCCCGGTTCGCGCTCCC TCCTTCCGTGTGTTGTCGTCTCTCGGGCGTCTAAGTACGC**T#**TAGGC-3′, reverse – 5′-ACCC CAGGGACTT*GAAGTAATAAG-3′.

### 2.2. Nucleosome assembly and purification

Nucleosomes were assembled using chicken donor chromatin without linker histone by salt dialysis as described [19]. The mononucleosomes were then gel-purified as described [20]. In-gel FRET analysis was performed using a Typhoon PhosphorImager. Fluorescence was excited in gel at 532 nm wavelength and recorded at 570–610 nm (for Cy3) and 650–700 nm (for Cy5) spectral regions.

### 2.3. spFRET measurements

Fluorescently labeled nucleosomes at 3 nM were incubated with 50 or 100 nM PARP-1 for 20 minutes in a buffer containing 20 mM Tris-HCl pH 7.9, 5 mM MgCl_2_, 150 mM KCl and 0.15 mM ZnCl_2_ at +25 °C in siliconized tubes. To induce poly(ADP)-ribosylation, nucleosomes were incubated with 50 nM PARP-1 for 20 min and further incubated with 2 or 4 µM NAD^+^ for 15 min. spFRET analysis was performed for 15 min using facilities and settings described previously [13]. spFRET measurements were repeated in at least two independent experiments. In each experiment, data from 700 to 7000 single nucleosomes were analyzed. Preservation of structures of nucleosomes and PARP-1-nucleosome complexes during the analysis was further verified by comparing the results of two consequent measurements.

Efficiency of FRET and its changes were characterized by calculating proximity ratio (E_PR_) for each single nucleosome:

$${\text{E}}_{\text{PR}}=\left(I_{5}-0.\text{19}\times I_{3}\right)/\left(I_{5}+0.\text{81}\times I_{3}\right)$$ (1)

where *I_5_* and *I_3_* are measured fluorescence intensities of Cy5 and Cy3, respectively, and factors 0.19 and 0.81 provide correction for the contribution of Cy3 fluorescence in the Cy5 detection channel (spectral cross-talk). E_PR_ values calculated for nucleosome samplings were presented as frequency distribution histograms and fitted by two Gaussians. Goodness of the fit (R^2^) varied from 0.84 to 0.99.

## 3. Results

### 3.1. The experimental approach for analysis of PARP-1-dependent changes in nucleosome structure

**Figure 1.**
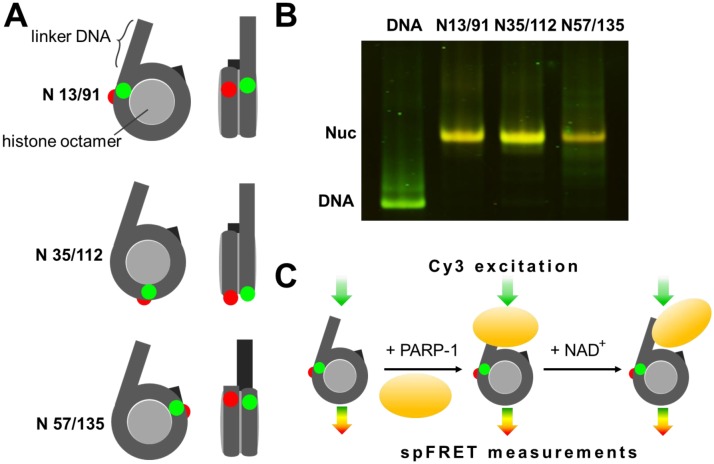
The experimental approach for analysis of PARP-1-dependent changes in nucleosome structure. **A.** Three types of mononucleosomes containing the single pair of Cy3 and Cy5 dyes in different positions on the nucleosomal DNA (the positions of Cy3 and Cy5 are shown by green and red circles, respectively). **B.** PAGE and in-gel FRET analysis of assembled nucleosomes and DNA template. Distributions of Cy3 and Cy5 fluorescence in a gel at a Cy3 excitation are shown in green and red, respectively. Yellow color (superposition of green and red colors) indicates a considerable FRET efficiency. **C.** Experimental approach. spFRET from nucleosomes was measured in the absence or presence of PARP-1 and subsequent addition of NAD^+^.

To study the effect of PARP-1 on the nucleosomal structure, spFRET microscopy experiments were conducted using three mononucleosomal templates; each nucleosome was labeled with a single pair of Cy3 and Cy5 fluorophores (Figure 1A). These labels were introduced in DNA based on known crystal structure of a nucleosome [21] to obtain efficient FRET between them in assembled nucleosomes without interfering with DNA structure or contacts between the DNA and core histones [13]–[16]. Labels were positioned into different parts of nucleosomal DNA: at +13 (Cy3) and +91 (Cy5) base pairs, relatively to the entry of linker DNA into nucleosome core (referred to as N 13/91), at positions +35 and +112 (N 57/135) and at +57 and +135 (N 57/135). In the assembled nucleosome, these positions are localized near the entry point of DNA into the nucleosome, near a contact between H2A-H2B histone dimers and close to the exit of DNA from nucleosome, respectively (Figure 1A).

Nucleosome assembly was carried out using chicken chromatin as a donor of core histones and a short DNA fragment containing nucleosome positioning sequence 603 [22] and additional terminal 20 bp linker. This linker provided a DNA end (“dsDNA break”) for PARP-1 binding to the nucleosome (Figure 1A); PARP-1 cannot bind to the other DNA end localized at the nucleosomal boundary [12],[23]. Quality of the assembly was estimated by native PAGE; the expected changes of the FRET signal in the nucleosomes as compared with histone-free DNA were observed in the gel (Figure 1B). Single-particle FRET measurements were conducted using gel-purified nucleosomes in solution under microscope. The Cy3 label was excited with 514.5 nm laser wavelength in single nucleosomes or complexes when they diffused freely through a small focal volume [13] (Figure 1C), and fluorescence intensities of both Cy3 (donor) and Cy5 (acceptor) dyes were measured. Proximity of the labels in single nucleosomes was characterized by calculating of so-called proximity ratio (E_PR_), and the frequency distribution of E_PR_ was plotted for each nucleosome sample. Absolute distances between labels were not calculated because of insufficient data about quantum yields and an instrumental factor.

### 3.2. PARP-1 induces structural changes in nucleosomal DNA

**Figure 2.**
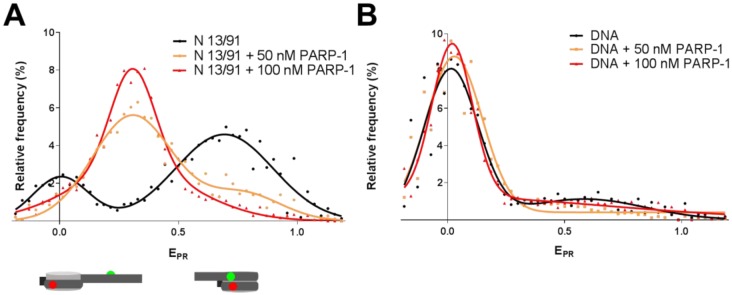
PARP-1 induces structural changes in nucleosomal DNA. **A.** spFRET analysis of PARP-1 binding to N 13/91 nucleosomes. Typical frequency distributions of E_PR_ are shown for N 13/91 nucleosomes before and after addition of 50 or 100 nM of PARP-1 (for quantitative and statistical data see Table S1). **B.** spFRET analysis of +13/+91-labeled DNA before and after addition of 50 or 100 nM of PARP-1.

In agreement with the previously published data [16], spFRET analysis revealed two populations of N 13/91 nucleosomes (Figure 2A, Table S1). A peak with the E_PR_ maximum at 0.69 corresponds to a major fraction of compact nucleosomes, while a peak with the E_PR_ maximum at 0.01 likely indicates the presence of a minor fraction of nucleosomes with partially unwrapped DNA. After addition of 50 nM PARP-1, the distribution of nucleosomes by E_PR_ is changed (Figure 2A) indicating formation of PARP-1-nucleosome complexes. These complexes are characterized by E_PR_ with a maximum at 0.31, while a shoulder in a higher E_PR_ region corresponds to PARP-1-free, more compact intact nucleosomes. Incomplete PARP-1 binding is observed most likely because the dissociation constant for PARP-1 complexes with similar nucleosome constructs is 85 nM [12]. In agreement with this proposal, a high E_PR_ shoulder disappears after increasing concentration of PARP-1 to 100 nM (Figure 2A). The low-E_PR_ peak at 0.01 is also diminished in the presence of PARP-1, suggesting that spontaneous DNA uncoiling from the octamer is diminished in the complex.

Domination of the single Gaussian peak in the frequency distribution of E_PR_ indicates formation of a single uniform population of nucleosome complexes with PARP-1. A shift of E_PR_ maximum from 0.69 to 0.31 shows that PARP-1 binding causes structural changes in nucleosomal DNA near the entrance of DNA into nucleosome, namely in the region, where DNA interacts with the H2A-H2B dimer (position +13) and H4-H2B interface (position +91). To evaluate a possibility that PARP-1 disrupts nucleosomes and forms complexes with histone-free DNA, the same experiments were conducted with DNA template used for nucleosome assembly (Figure 2B). In more extended histone-free DNA, the Cy3 and Cy5 labels are positioned far from each other and no FRET occurs (maximum of E_PR_ is 0.01, Figure 2B, Table S1). The frequency distribution of E_PR_, which is observed for the histone-free DNA, is minimally affected by PARP-1 (Figure 2B).

In summary, binding PARP-1 to the N 13/91 nucleosome causes considerable structural changes in nucleosomal DNA that are accompanied by an increase in the distance between the labels introduced near the entrance of DNA into a nucleosome, indicating that gyres of nucleosomal DNA are coming apart and, at the same time, by diminished spontaneous DNA uncoiling from the octamer in the complex, suggesting that the DNA end has a lower mobility in the PARP-1-nucleosome complex. These observations, taken together, suggest that PARP-1 causes a mobility of the end of nucleosomal DNA, but, at the same time, induces uncoiling of nucleosomal DNA together with histones.

### 3.3. PARP-1 induces similar structural changes in different regions of nucleosomal DNA

**Figure 3.**
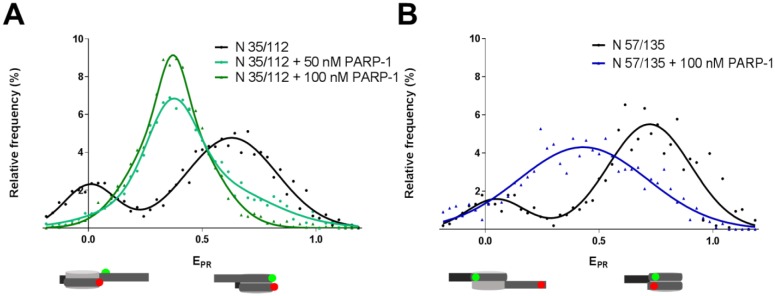
PARP-1 induces similar structural changes in different regions of nucleosomal DNA. spFRET analysis of PARP-1 binding to N 35/112 (A) and N 57/135 nucleosomes (B). Typical frequency distributions of E_PR_ are shown for nucleosomes before and after incubation in the presence of 50 or 100 nM of PARP-1 (for quantitative and statistical data see Table S1).

To evaluate how PARP-1 affects other parts of nucleosomal DNA, N 35/112 and N 57/135 nucleosomes were analyzed (Figure 3). The N 13/91 (Figure 2A) and N 35/112 nucleosomes (Figure 3A) are characterized by similar frequency distributions of E_PR_ (see Table S1 for the statistical data). Although labels in the nucleosomes N 35/112 were positioned far from the extending DNA end (PARP-1 target), FRET between theses labels was significantly affected by PARP-1 binding. The maximum of the main E_PR_ peak was shifted from 0.63 to 0.37, indicating to reorganization of nucleosomal DNA structure near the interface between the H2A/H2B dimers and H3/H4 tetramers (position +35) and/or H3/H4 tetramers (position +112) that resulted in the increase in the inter-label distance.

N 57/135 nucleosomes were also characterized by a bimodal distribution of E_PR_ (Figure 3B and Table S1). In the presence of 100 nM PARP-1, a broad E_PR_ distribution was formed with a maximum at 0.43. This broadening could be explained by an increased mobility of nucleosomal DNA localized near the position +135 in the PARP-1-nucleosome complex.

For every combination of the labels, the shift of the main peak from higher to intermediate E_PR_ values was observed. In the case of N 13/91 and N 35/112 the shift was accompanied by a significant decrease in the height of the low-E_PR_ peak (Figures 2 and 3). Taken together, the data indicate that PARP-1 can partially and similarly uncoil different regions of nucleosomal DNA, and, at the same time, can restrict mobilities of the +13, +35 and +112 regions of nucleosomal DNA.

### 3.4. PARP-1-induced changes in nucleosome structure are reversed after PARP-1 automodification

**Figure 4.**
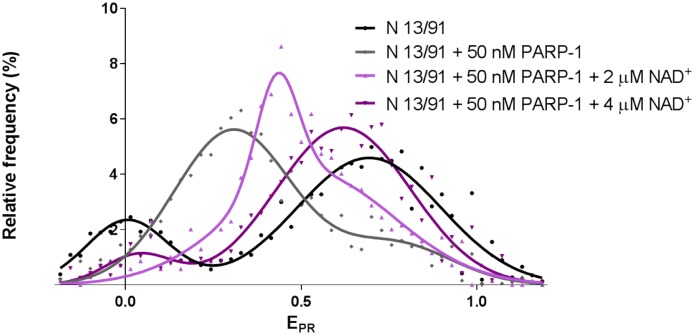
PARP-1-induced changes in nucleosome structure are reversed after PARP-1 automodification. spFRET analysis of PARP-1 automodification in the complex with nucleosomes N 13/91 after addition of NAD^+^. Typical frequency distributions of E_PR_ are shown (for quantitative and statistical data see Table S1).

To elucidate how activation of the enzymatic activity of PARP-1 affects the structure of the PARP-1-nucleosome complex, pre-formed PARP-1 complexes with N 13/91 nucleosomes were incubated in the presence of different concentrations of NAD^+^. DNA-bound PARP-1 is activated, auto-poly(ADP)-ribosylated in the presence of NAD^+^ and loses its capability to interact with damaged DNA and nucleosomes [23],[24]. Therefore, it was expected that nucleosomal E_PR_ distribution would be recovered in the presence of NAD^+^. spFRET analysis revealed that incubation of the PARP-1-nucleosome complex in the presence of 2 or 4 µM NAD^+^ results in a progressive, stepwise shift of the mean value of E_PR_ peak from 0.31 to 0.43 or to 0.62, respectively (Figure 4 and Table S1). In the presence of 4 µM NAD^+^, the main E_PR_ maximum (0.62) approaches the value, which is a characteristic of free nucleosomes (0.69), suggesting that nucleosome structure is almost completely recovered. Since the principal NAD^+^-dependent reaction is PARP-1 automodification [25], the data indicate that after partial automodification (i.e., at 2 µM NAD^+^) PARP-1 remains bound to nucleosomes and forms a discrete intermediate PARP-1-nucleosome complex.

Incomplete reversal of the nucleosomal E_PR_ distributions at 4 µM NAD^+^ is likely explained by incomplete automodification of PARP-1 that remains bound to the nucleosome. Alternatively, poly(ADP)-ribosylation of core histones [8] prevents complete recovery of the nucleosomal structure.

## 4. Discussion

Our spFRET experiments suggest that PARP-1 binds to a nucleosome and induces disturbance of different regions of nucleosomal DNA: near the entrance/exit of DNA into/from a nucleosome, and in the region positioned ∼35 bp from the boundaries of nucleosomal DNA (Figures 2 and 3). This uncoiling of nucleosomal DNA is accompanied by a reduced mobilities of the +13, +35 and +112 regions of nucleosomal DNA (Figures 2 and 3). PARP-1 automodification (self-PARylation) is accompanied by formation of an intermediate complex, and eventually leads to nearly complete recovery of the initial structure of nucleosome (Figure 4). Thus spFRET is a sensitive method for analysis of PARP-1-induced changes in chromatin structure that could also be used for analysis of PARP-1 inhibition by various compounds.

**Figure 5.**
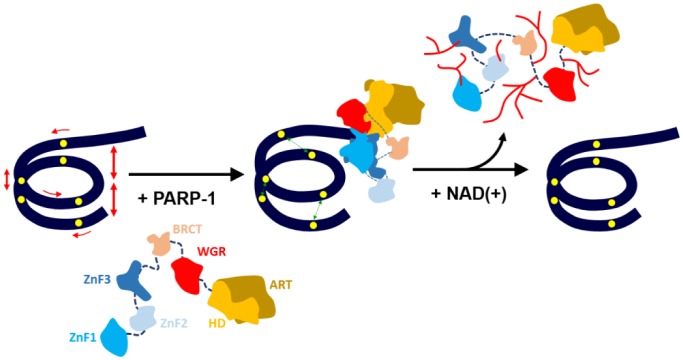
The model of PARP-1-induced conformational changes in nucleosomal DNA in the vicinity of double-strand DNA break. DNA is tightly wrapped around histone octamer, but there are fluctuations in the structure of the double helix, especially in an area near DNA entering or exiting the nucleosome core. Unmodified PARP-1 is inactive and its domains (shown by different colors) are connected together by flexible linkers [26]. When PARP-1 binds to a DNA damage or available DNA end in the vicinity of a nucleosome, it forms a compact structure [7], which induces activation of PARP-1 and partial unwrapping of nucleosomal DNA. This nucleosome unfolding is accompanied by a reduced mobilities of the +13, +35 and +112 regions of nucleosomal DNA and possibly by destabilization of the intranucleosomal interactions between core histones. In the presence of NAD^+^ PARP-1 is automodified and released from the nucleosome; PARP-1 release is accompanied by spontaneous recoiling of nucleosomal DNA. Yellow circles indicate positions of the pairs of fluorescent dyes on nucleosomal DNA.

PARP-1 binds to nucleosomes having one linker DNA with an exposed double-strand break with stoichiometry of one PARP-1 molecule per nucleosome [12]. Rearrangements in the enzyme structure after binding to a double-strand DNA break [7] make HD subdomain unstable, resulting in activation of the catalytic center of PARP-1 [26],[27]. If PARP-1 is bound in the vicinity of a nucleosome, activated PARP-1 can also induce a considerable, partial and reversible disturbance of nucleosomal DNA structure (Figure 5). Similar, although less pronounced changes of nucleosomal structure have been observed after acetylation of core histones and DNA methylation in a nucleosome [28],[29]. Much more dramatic uncoiling of nucleosomal DNA together with the associated core histones was observed in the complex between yFACT and a nucleosome [16]. It is possible that yFACT and PARP-1 induce conformational changes in nucleosomal DNA of similar nature, but different magnitude.

Nucleosome structure can be considerably changed during various processes, such as transcription [30]–[32] and protein binding to nucleosomal DNA [33]. These conformational changes include: (i) DNA unwrapping from an intact octamer; (ii) DNA unwrapping accompanied by opening of the (H2A-H2B) dimer/(H3-H4)_2_ tetramer interface; (iii) DNA unwrapping with complete octamer disassembly and (iv) the unwrapping involving opening of the (H3-H4)_2_ tetramer [34]–[36]. Since different regions of nucleosomal DNA are uncoiled in the PARP-1-nucleosome complex to a similar degree (Figure 5), the global change in the nucleosome structure involving structural changes in the entire histone octamer likely occurs. The nature and extent of these conformational changes in nucleosome structure remain to be determined.

Detection and repair of double-strand breaks in cells require multi-step chromatin remodeling. Thus p400/Tip60 chromatin remodeler induces exchange of histones H2A to H2A.Z onto nucleosomes at the break, which is important for downstream acetylation of H4 histone and maintenance of less compact chromatin structure in the break region [37]. PARP-1-induced DNA uncoiling could facilitate the histone exchange and/or displacement during the chromatin remodeling, before PARP-1 leaves the DNA break after automodification. It is also possible that the stable PARP-1-nucleosome complexes could be formed at transcription start sites [4].

## 5. Conclusions

Nucleosome structure can be considerably and reversibly unfolded after PARP-1 binding. These changes include transient and partial uncoiling of nucleosomal DNA along its entire length. These PARP-1-dependent changes in nucleosome structure are nearly completely reversed after PARP-1 eviction due to its auto-poly(ADP)-ribosylation.
