# Supplementary material for: Unfolding of core nucleosomes by PARP-1 revealed by spFRET microscopy
Source: AIMS Genet. 2017 Jan 5;4(1):21–31. doi: 10.3934/genet.2017.1.21 (PMC5552189; doi:10.3934/genet.2017.1.21)
Supplement: Supplementary file 1 [file genetics-04-01-021-s01.pdf]

AIMS Genetics

AIMS Genetics, 4(1): 21-31.
DOI: 10.3934/genet.2017.1.21
Received: 08 November 2016
Accepted: 29 December 2016
Published: 05 January 2017

http://www.aimspress.com/journal/Genetics

Research article

Unfolding of core nucleosomes by PARP-1 revealed by spFRET microscopy

Daniel Sultanov[1,2], Nadezhda Gerasimova[1], Kseniya Kudryashova[1,3], Natalya Maluchenko[1], Elena Kotova[2], Marie-France Langelier[4], John Pascal[4], Mikhail Kirpichnikov[2], Alexey Feofanov[1,3]* and Vasily Studitsky[1,2,]*

[1] Biology Faculty, Lomonosov Moscow State University, Moscow, 119992, Russia

[2] Fox Chase Cancer Center, Philadelphia, PA, 19111-2497, USA

[3] Shemyakin-Ovchinnikov Institute of Bioorganic Chemistry of Russian Academy of Sciences, 117997 Moscow, Russia

[4] Department of Biochemistry and Molecular Medicine, Université de Montréal, 2900 Boulevard, Edouard-Montpetit, Montréal, QC H3T 1J4, Canada

* **Correspondence:** Email: avfeofanov@yandex.ru, vasily.studitsky@fccc.edu; Tel: +215-214-7134; Fax: +215-728-3574.

Abstract: DNA accessibility to various protein complexes is essential for various processes in the cell and is affected by nucleosome structure and dynamics. Protein factor PARP-1 (poly(ADP-ribose) polymerase 1) increases the accessibility of DNA in chromatin to repair proteins and transcriptional machinery, but the mechanism and extent of this chromatin reorganization are unknown. Here we report on the effects of PARP-1 on single nucleosomes revealed by spFRET (single-particle Förster Resonance Energy Transfer) microscopy. PARP-1 binding to a double-strand break in the vicinity of a nucleosome results in a significant increase of the distance between the adjacent gyres of nucleosomal DNA. This partial uncoiling of the entire nucleosomal DNA occurs without apparent loss of histones and is reversed after poly(ADP)-ribosylation of PARP-1. Thus PARP-1-nucleosome interactions result in reversible, partial uncoiling of the entire nucleosomal DNA.

Keywords: PARP-1 protein; DNA repair; chromatin structure; nucleosome; DNA-histone interactions

Supplementary

Table S1. Quantitative analysis of spFRET data.

	Sample size (number of single particle events)	Goodness of fit, R^2	Mean value of E_{PR} peak ± SE	
			Mean 1	Mean 2
DNA	3647	0.89	0.01 ± 0.01	0.58 ± 0.09
DNA +50 nM PARP-1	8338	0.93	0.03 ± 0.02	-
DNA + 100 nM PARP-1	2732	0.98	0.02 ± 0.01	
N 13/91	6677	0.88	0.01± 0.01	0.69 ± 0.02
N 13/91 + 50 nM PARP-1	1845	0.95	0.31 ± 0.01	0.77 + 0.04
N 13/91 + 100 nM PARP-1	1977	0.98	0.31 ± 0.01	-
N 13/91 + 50 nM PARP-1 + 2 µM NAD^+	731	0.94	0.43 ± 0.01	0.53 ± 0.02
N 13/91 + 50 nM PARP-1 + 4 µM NAD^+	1216	0.93	0.04 ± 0.03	0.62 ± 0.01
N 35/112	6044	0.95	0.01 ± 0.01	0.63 ± 0.02
N 35/112 + 50 nM PARP-1	4648	0.98	0.37 ± 0.01	0.47 ± 0.02
N 35/112 + 100 nM PARP-1	2877	0.99	0.37 ± 0.01	-
N 57/135	1547	0.84	0.05 ± 0.03	0.72 ± 0.04
N 57/135 + 100 nM PARP-1	1195	0.89	0.43 ± 0.01	-
